# Linking Psychological Capital and Behavioral Support for Change: The Roles of Openness to Change and Climate for Innovation

**DOI:** 10.3389/fpsyg.2021.612149

**Published:** 2021-08-03

**Authors:** Jieqiong Liu

**Affiliations:** School of Management, Guizhou University, Guiyang, China

**Keywords:** psychological capital, openness to change, climate for innovation, behavioral support for change, transactional stress theory

## Abstract

Employees’ behavioral support for change is seen as a key factor for the success of organizational changes. Given the assumed importance, the study developed a multilevel conditional mediation model and examined whether psychological capital influences behavioral support for change and if so, how the effect occurs. Results from a sample of 69 team leaders and 319 followers showed that the positive impact of psychological capital on openness to change was stronger in teams high in climate for innovation and that the mediating role played by openness to change in the relationship of psychological capital to behavioral support for change was significant when climate for innovation was high.

## Introduction

Organizations today face highly competitive environments and unpredictable technological advances, which results in the increasing importance attached to successful organizational changes. However, many changes often failed to deliver the expected outcomes (Van den Heuvel et al., 2014). A key factor for the success of changes is behavioral support for change, which refers to individuals’ demonstration of extreme enthusiasm for a change by going above and beyond what is formally required to ensure the success of the change and promoting the change to others (Herscovitch and Meyer, 2002). Yet employees are often reluctant to support changes because organizational change (e.g., changes in job roles, expectations, and working relationships) increases job demands such as new routines, skills, responsibilities and other emotional demands; thus, employees often regard organizational change as stressors (Lee et al., 2017). In this regard, research on factors that facilitate behavioral support for change will be beneficial for practitioners to cope with organizational change and improve its success.

Transactional stress theory suggests that employees appraise stressors as either challenges or threats (Lazarus and Folkman, 1984). When stressors are perceived as challenges, the stressors will lead to positive outcomes; if they are perceived as threats, negative outcomes may occur, indicating that responses to stressors vary as a function of individual differences (Lazarus and Folkman, 1984). Carmeli and Schaubroeck (2007) argued that employees will only see a demand (a specific type of job stressor) as challenging when they feel capable of dealing with it. In this study, we focused on psychological capital (PsyCap), because PsyCap is an employee’s positive psychological state of development based on four positive psychological abilities, namely, hope, optimism, resilience and self-efficacy (Luthans et al., 2007); moreover, previous studies have indicated that PsyCap enables employees to better cope with stressful events (e.g., organizational change), as a result, interpret them in a positive way and respond with positive behaviors (e.g., Avey et al., 2009; Roberts et al., 2011). More specifically, the study aimed to explore whether high-PsyCap employees undergoing organizational changes perceive the changes as challenges and show active behavioral support and, if so, how the effect occurs and under what conditions PsyCap has a stronger explanatory effect on outcomes.

Drawing on transactional stress theory, appraisals of changes as challenges or threats are associated to positive and negative emotional experiences (Lazarus and Folkman, 1984), which, in turn, influence how employees cope with stressors. Consistent with the assumption underlying this theory, Dong et al. (2014) found that affective experiences mediate the relationship between developmental job experience and work outcomes. As such, we argue that emotional response to change may be a potential mediator linking PysCap and behavioral support for change. This study focused on the mediating role of openness to change, because it is an important but often neglected emotion-laden construct in organizational change research (Bouckenooghe, 2010).

The interactionist perspective indicates that individual behavior is a result of interplay between environment and individual (Wu et al., 2014). That is, individual behavior can’t be explained better if overlooking individual factors or the context where it occurs (Liden and Antonakis, 2009). Therefore, drawing on this perspective, this study takes into account the contextual factors and further explores the boundary conditions for PsyCap’s effect. Previous research has indicated that context might play an important role for the effectiveness of PysCap (Walumbwa et al., 2010; Cai et al., 2019; Xu et al., 2020). For example, the positive effect of PysCap on job performance has been found to be stronger under the conditions of high service climate (Walumbwa et al., 2010). Cai et al. (2019) found that the impact of PsyCap on employee creativity was moderated by supervisor support for creativity and job characteristics (e.g., autonomy). The perceptions of change stem from employees’ perceptions of the work situations (Martin et al., 2005). As such, creating a climate conductive to changes (e.g., climate for innovation) may be particularly helpful in improving employees’ appraisals of change because the supportive climate sends a signal that behavioral support for change is expected and rewarded. Therefore, this study examined climate for innovation as a moderator of PysCap’s effectiveness and proposed that PsyCap has stronger effects on openness to change and subsequently behavioral support for change when climate for innovation is high.

Overall, drawing on transactional stress theory and the person-context interaction perspective, this study integrated openness to change and climate for innovation as mediating and moderating mechanisms into a multilevel model that explained how and when PsyCap positively impacts behavioral support for change. To this end, we used a two-wave data including 69 team leaders and 319 followers and empirically examined the theoretical model.

## Theory and Hypotheses

### PsyCap and Behavioral Support for Change

Organizational changes may increase employees’ stress by placing them in dynamic work environments where they must think and act in new ways, and learn new routines and skills. Research shows that the same stressor occurs to two different employees and causes negative outcomes in one of them but not in the other (Vakola, 2014). Transactional stress theory may provide a useful theoretical foundation for explaining the inconsistent findings in the literature. Transactional stress theory argues that employees appraise stressors as either challenges or threats; challenges lead to positive outcomes while threats are related to negative outcomes (Lazarus and Folkman, 1984). According to this theory, although all demands are stressful, a key determinant of whether demands result in negative outcomes is not the extent of demands but the type of demands. PsyCap is argued to have an inherent proactive coping mechanism and goal orientation that may help employees effectively cope with stressors (Abbas et al., 2012). High-PsyCap employees expect good things to happen at work and trust their abilities to create future success (Avey et al., 2011). Consequently, they tend to perceive stressors in a positive way and respond with positive behaviors (Roberts et al., 2011). Based on the above, we proposed that employees with high PsyCap may appraise changes as challenges and respond with behavioral support for change. Although this proposition has not been directly tested, previous studies provided indirect support because it has found that employees high in PsyCap trend to interpret stressful events in a positive way, and thus, respond more positively (e.g., Avey et al., 2009; Roberts et al., 2011; Abbas and Raja, 2015; Siu et al., 2015). Therefore, we hypothesize:

**Hypothesis1**: PsyCap is positively related to behavioral support for change.

### The Mediating Role of Openness to Change

Transactional stress theory posits stressors, appraised as either challenges or threats, can evoke emotional experiences, thereby influencing how employees deal with those stressors (Lazarus and Folkman, 1984). In particular, the theory suggests that challenge stressors, because they are appraised as potentially fostering personal gains or growth, evoke positive emotions and problem-focused coping behaviors, while hindering stressors, because they are appraised as potentially thwarting personal gains or growth, evoke negative emotions and emotion-focused coping behaviors (LePine et al., 2005). As such, PsyCap promotes behavioral support for change, at least in part because employees high in PsyCap appraise changes as challenges that potentially promote personal gains or growth, thereby triggering positive emotional responses to change. This study attempted to examine whether the effect of PsyCap on behavioral support for change is mediated by openness to change.

Openness to change is a vital emotional state that is conceptualized as positive affect about the potential outcomes of the change and willingness to support the change (Miller et al., 1994). According to transactional stress theory, employees tend to experience positive feelings when they appraise stressors as challenges (Lazarus and Folkman, 1984). The appraisal of challenges is related to beliefs about the relationship between effort made to deal with certain demand and the probability of success in meeting the demand (expectancy) and about the relationship between success in meeting the demand and obtaining valued outcomes (instrumentality; LePine et al., 2005). High-PsyCap employees believe that they can deal with change-related challenges, can generate alternative pathways to overcome obstacles and achieve goals, have positive expectations about future outcomes, and exhibit flexibility and persistence when faced with hardship (O’Donohue et al., 2015). Therefore, they believe that expending effort will allow them to successfully meet change-related demands (expectancy). Moreover, they also further believe that success in meeting these demands can bring in beneficial consequences, such as organizational support, promotion opportunities and increased salaries (instrumentality), because positive coping behaviors are expected and desired by their organizations that are implementing change initiatives. In addition to organizational inducements, high-PsyCap employees may also develop new skills and achieve personal growth during the process of expending effort to meet demands, because there is evidence indicating that challenging demands provide employees with opportunities to fully apply their skills and stretch their capabilities (Zhou et al., 2011). Taken together, we argued that employees with high PsyCap could meet with change-related demands and achieve personal growth or gains, thereby enhancing openness to change.

Lazarus and Folkman (1984) suggested that positive emotional experiences resulting from challenge appraisals of stressors promote positive coping behaviors. As such, openness to change evoked by PsyCap may positively influence behavioral support for change. Based on its definition, openness to change can be characterized by two related factors: anticipated benefits of change and a strong desire to support the change, both of which are well-established motivating forces encouraging behavioral support for change. First, the literature has indicated that behavioral support for change is related to the perception of anticipated benefits of the change (Oreg et al., 2011). When changes are perceived as personally beneficial, employees will exert efforts to contribute to the success of changes. The second characteristic is a strong desire to support the change, which represents strong change-supportive intentions. The behavioral intentions have been identified as important and most proximal antecedents of behavioral support for change (Straatmann et al., 2018). Therefore, we proposed that openness to change has a positive effect on behavioral support for change.

To sum up, the discussion above indicated that PsyCap influences openness to change, thereby contributing to behavioral support for change. Therefore, we hypothesize:

**Hypothesis2:** Openness to change mediates the relationship between PsyCap and behavioral support for change.

### The Moderating Role of Climate for Innovation

Climate for innovation refers to a set of shared perceptions about policies, practices, and procedures that are advocated by an organization and that encourage employees to take initiatives, and explore and develop new ideas. A better understanding of organizational climate should be critical for the management of change, because the climate shapes employees’ perceptions regarding the change process itself (Martin et al., 2005). However, existing knowledge on the effect of climate on changes is still limited (Rogiest et al., 2016). Some literature indicates that the strength of the relationships of PsyCap to its outcomes depends on the level of climate. For example, the effect of PsyCap on job performance is moderated by service climate (Walumbwa et al., 2010). Therefore, this study attempted to examine climate for innovation as a moderator of the PsyCap-openness to change relationship.

A plausible explanation for the moderating effect of climate for innovation is that the climate provides employees with information about expected and rewarded behaviors, thereby enhancing the beliefs caused by PysCap that if certain demands are met, valued results will occur. Specifically, when employees perceive climate for innovation is high, they have come to recognize that behavioral support for change is highly expected, desired and rewarded by management to successfully implement change initiatives. Such perceptions may contribute to high-PsyCap employees’ judgments that they can successfully meet change-related demands by expending their effort and that by doing so they can achieve corresponding rewards. Therefore, in such a climate, employees high in PsyCap are more likely to show higher openness to change.

Conversely, when climate for innovation is low, the work context signals to employees that behavioral support for change is discouraged or unappreciated, thereby potentially weakening the positive relationship between success in meeting demands and obtaining valued outcomes. That is, in the climate, employees with high PsyCap may not see the value or rewards for their efforts, even though they believe that they can meet these demands by exerting effort. Furthermore, the unsupportive climate, due to its less job autonomy, may constrain these employees’ ability to engage in successful enactive mastery experiences, thereby potentially thwarting personal growth (Jiang and Gu, 2015). Therefore, PsyCap is less functional in the climate. Therefore, we hypothesize:

**Hypothesis3**: Climate for innovation moderates the relationship between PsyCap and openness to change, such that the relationship is stronger in teams with high rather than low climate for innovation.

**FIGURE 1:**
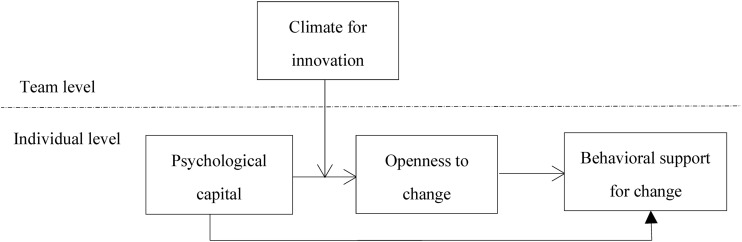
The conceptual model.

According to the arguments above, PsyCap has an indirect effect on behavioral support for change via openness to change (hypothesis 2) and the relationship of PsyCap to openness to change is moderated by climate for innovation (hypothesis 3). Combining hypothesis 2, and 3, we further posit a moderated mediation model shown in Figure 1, namely, the mediating role played by openness to change in the relationship of PsyCap to behavioral support for change depends on climate for innovation. Therefore, we hypothesize:

**Hypothesis4**: Climate for innovation moderates the mediating effect of openness to change on the relationship of PsyCap to behavioral support for change, such that mediating effect is stronger in teams with high rather than low climate for innovation.

## Materials and Methods

### Participants and Procedure

The study was conducted in a large state-controlled pharmaceutical company in China that underwent organizational changes including the introduction of new production machinery and performance appraisal system, departmental merges, relocation of employees, modifications of established working relationships and methods, and improving professionalization through training program. The goals of these change initiatives were to increase flexibility, operation and production efficiency and finally remain competitive in a dynamic environment. With the support of the change management team, the survey was distributed to 432 employees nested within 87 teams through their leaders. Participation in the survey was voluntary and anonymous. It is a two-wave study with six-month interval. The first survey was conducted at the beginning of the change initiatives and to measure PsyCap, climate for innovation, and control variables. The second survey was conducted about six months later to measure openness to change and behavioral support for change. The respondents who completed the two surveys were 319 employees comprising 69 teams (overall response rate = 74%). Among of these, 46.7% were female, their mean age was 30.57 years (*SD* = 7.13), and average tenure was 5.05 years (*SD* = 5.18).

### Measures

For all focus variables, respondents rated their agreement on a 7-point Likert-type scale ranging from 1 (strongly disagree) to 7 (strongly agree).

#### PsyCap (α = 0.87)

We measured PsyCap with a 24-item scale provided by Luthans et al. (2007). Example items include: self-efficacy— “I feel confident in analyzing a long-term problem to find a solution”; hope— “I can think of many ways to reach my current work goals”; resilience— “I can get through difficult times at work because I have experienced difficulties before” and optimism— “I always look on the bright side of things regarding my job.”

#### Openness to Change (α = 0.72)

We measured openness to change with Wanberg and Banas’s (2000) 7-item scale. Example items include “I am quite reluctant to accommodate and incorporate these changes into my work.”

#### Climate for Innovation (α = 0.82)

We measured climate for innovation with Kivimaki and Elovainio’s (1999) 14-item scale. It has four dimensions, including participative safety (4 items), vision (4 items), task orientation (3 items) and support for innovation (3 items). Example items include: participatory safety— “People really attempt to share information”; vision— “People are agreement with the objectives”; task orientation— “People make critical appraisal of weaknesses” and support for innovation— “People cooperate with each other in developing and applying ideas.”

#### Behavioral Support for Change (α = 0.67)

In accordance to Seo et al. (2012), behavioral support for change was measured using four items selected from the scale of Herscovitch and Meyer’s (2002) that reflect most active forms of supporting change initiative. An example item is “I’ve put in a good deal of effort in trying to do what I can to make the transition succeed.”

### Control Variables

Following prior research (e.g., Choi, 2007), gender, age, education, organizational tenure, hierarchical position, and team size were used as control variables in our analyses.

## Analytical Approach

Before the tests of hypotheses, we computed the intraclass correlation (ICC1) values to assess the necessity of cross-level analysis. The ICC1 values for openness to change and behavioral support for change were 0.61 and 0.48, indicating that 61% of the variance in openness to change and 48% of the variance in behavioral support for change resided between teams, respectively, thereby justifying a cross-level analysis for hypothesis test. Additionally, the construct is considered as a team variable in this study, thus, within-group agreement (RWG), ICC1, and ICC2 were tested to determine whether the aggregation was appropriate. The mean RWG, ICC1, and ICC2 values were 0.98, 0.71, and 0.92, respectively, supporting the aggregation of climate for innovation.

### Multilevel Confirmatory Factor Analysis

We used Mplus 7.4 to conduct multilevel confirmatory factor analyses to examine construct distinctiveness. As shown in Table 1, the four-factor model fits the data well [χ^2^(621) = 843.24, RMSEA = 0.03, SRMR(Within/Between) = 0.054/0.058, CFI = 0.94, TLI = 0.93], supporting the construct distinctiveness between PsyCap, openness to change, climate for innovation, and behavioral support for change.

**TABLE 1:** Alternative model test results for the study variables.

Model	χ^2^	df	Δχ2	RMSEA	SRMR (Within/Between)	CFI	TLI
four-factor (expected model)	843.24	621		0.03	0.054/0.058	0.94	0.93
three-factor (psychological capital and openness to change merged)	1105.40	623	262.16***	0.05	0.070/0.058	0.86	0.85
two-factor (psychological capital, openness to change and behavioral support for change merged)	1238.91	624	395.67***	0.06	0.073/0.058	0.82	0.81

***P < 0.001.

### Descriptive Statistics

Table 2 showed descriptive statistics and coefficients of correlations among all variables. PsyCap had a positive correlation with openness to change (*r* = *0.44, p* < *0.01*), and behavioral support for change (*r* = *0.36, p* < *0.01*). Openness to change had a positive correlation with behavioral support for change (*r* = *0.44, p* < *0.01*).

**TABLE 2:** Means, standard deviations, and correlations for all variables.

Variables	Means	SD	1	2	3	4	5	6	7	8	9
1 Gender	0.47	0.50	–	–	–						
2 Age	30.57	7.13	–0.10	–	–						
3 Education	3.08	0.81	−0.14*	0.19*	–						
4 Organizational tenure	5.05	5.18	–0.05	0.78**	–0.04						
5 Hierarchical position	2.73	0.79	0.12*	−0.49**	−0.15**	−0.35**					
6 Team size	5.08	1.62	0.06	–0.08	0.06	–0.01	–0.09				
7 Psychological capital	3.73	0.39	0.04	0.03	0.01	0.09	–0.64	0.05			
8 Climate for innovation	3.87	0.32	–0.08	0.05	0.00	0.11	−0.12*	0.03	0.63**		
9 Openness to change	3.53	0.46	0.04	0.11*	0.02	0.10	–0.02	0.01	0.44**	0.44**	
10 Behavioral support for change	3.61	0.52	–0.01	0.11	–0.02	0.07	−0.12*	0.03	0.36**	0.39**	0.44**

Hierarchical position was measured by number from 1 to 4 (1-associate, 2-assistant manager, 3-manager, 4-executive). Team size ranged from 3 to 10 members. **p < 0.01; *p < 0.05.

### Hypothesis Tests

**FIGURE 2:**
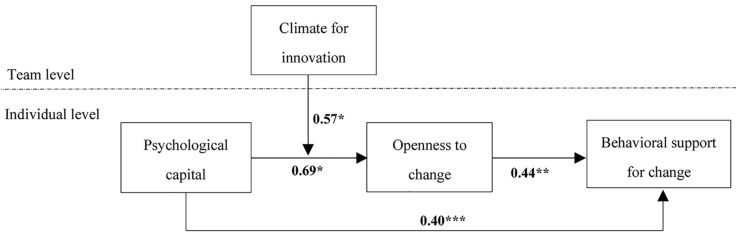
Moderated mediation model path coefficients. For the sake of parsimony, we did not present the influences of control variables on behavioral support for change. Interested readers may contact the first author for estimates of these influences. ***p < 0.001;*p < 0.05.

In our research, PsyCap, openness to change and behavioral support for change were individual-level variables and climate for innovation was a team-level variable. Therefore, we conducted multilevel analyses with Mplus 7.4 to test the proposed hypotheses. PsyCap was group-mean centered and climate for innovation was grand-mean centered before entering them into the analysis. The multilevel modeling results in Figure 2 showed that PsyCap was positively related to behavioral support for change (*b* = *0.40, p* < *0.001*), showing support for hypothesis1. Additionally, the path coefficients were significant from PsyCap to openness to change (*b* = *0.69, p* < *0.05*) and from openness to change to behavioral support for change (*b* = *0.44, p* < *0.01*). We used the Monte Carlo simulation procedure with 20,000 replications to construct confidence intervals (CI) for indirect influence. The results showed PsyCap had indirect influence on behavioral support for change via openness to change (b = 0.30, 95% CI [0.015, 0.636]), showing support for hypothesis 2.

**FIGURE 3:**
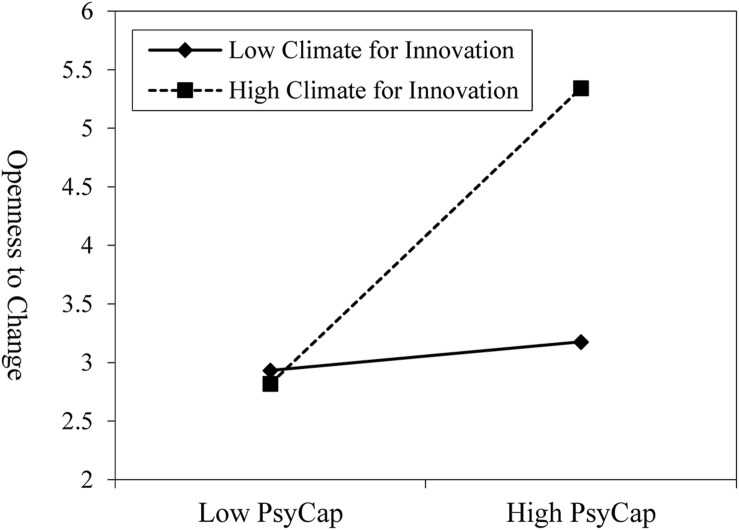
Moderating effect of climate for innovation on the relationship between PsyCap and openness to change.

Figure 2 also showed that the relationship of PsyCap to openness to change was moderated by climate for innovation (*b* = *0.57, p* < *0.05*). In order to understand the nature of the moderation, we conducted a simple slope analysis. Figure 3 showed that when climate for innovation was high rather than low, PsyCap has a stronger effect on openness to change. Therefore, hypothesis 3 was supported.

The Monte Carlo simulation procedure with 20,000 replications was conducted to test hypothesis 4. The results showed that when climate for innovation was high (*b* = *0.38, SE* = *0.17, 95% CI* [*0.06, 0.76*]), the moderating effect of openness to change on the relationship of PsyCap to behavioral support for change was significant, and it became non-significant when climate for innovation was low (*b* = *0.22, SE* = *0.14, 95% CI* [*−0.06, 0.56*]). Therefore, hypothesis 4 was supported.

## Discussion

The study developed a multilevel conditional mediation model and examined whether the mediating role played by openness to change in the relationship of PsyCap to behavioral support for change depends on climate for innovation. Consistent with the proposed hypotheses, the results showed that openness to change was a key mediating mechanism through which PsyCap positively influenced behavioral support for change. Additionally, we also found that high level of climate for innovation strengthened the direct effect of PsyCap on openness to change, and the mediating role played by openness to change in the relationship of PsyCap to behavioral support for change.

### Theoretical Implications

The findings make three significant contributions to the literature. First, our research contributes to the organizational change literature by proposing and confirming the effect of PsyCap on behavioral support for change. Organizational change research has highlighted the importance of considering individual factors because they influence how employees cope with changes (Oreg et al., 2011). These studies appear particularly valuable for both understanding the heterogeneity in responses to change and identifying more effective interventions to improve the success of changes. However, in many cases, individual differences, such as personality traits and demographic, are of limited use for the successful management of changes, because they are very stable and difficult to change (Straatmann et al., 2018). This study extends this line of research by introducing PsyCap that is open to development and demonstrating its importance for improving behavioral support for change.

Second, our study further contributes to the research on openness to change by identifying openness to change as a mediator linking PsyCap and behavioral support for change. Given the importance of emotional responses to change for change outcomes, previous studies have mostly focused on several important emotional responses (e.g., readiness for change, affective commitment to change), and what has been missing from research attention is openness to change (Bouckenooghe, 2010). These constructs represent individual positive cognitive evaluation of organizational change, and thus, they are similar (Choi, 2011). However, they also have distinct meanings and focuses, which may provide different implications for research and practice (Choi, 2011). Consequently, better understanding of the role of openness to change in influencing change-related outcomes is needed. Drawing on transactional stress theory, we find that employee with high PsyCap tend to appraise changes as challenges, thereby experiencing openness to change and subsequently showing behavioral support for change.

Third, this study also extends the psychological capital literature by answering the call of Newman et al. (2014) to examine boundary conditions for the effectiveness of PsyCap. PsyCap has been established a strong predictor of attitudes and behaviors. Yet, very little research has focused on the potential boundary conditions that either promote or hinder the positive effects of PsyCap (Walumbwa et al., 2010; Newman et al., 2014). Limited knowledge of boundary conditions inhibits our understanding of the usefulness and application of PsyCap theory in the workplace. Addressing this research gap, this study takes the person-context interaction perspective and suggest that high level of climate for innovation strengthens the positive effect of PsyCap on openness to change.

### Practical Implications

From a practical perspective, our findings indicate the important role of PsyCap in promoting openness to change and behavioral support for change. Moreover, PsyCap is considered as malleable, open-to-development, and can be improved by developmental interventions (Luthans et al., 2010). Therefore, organizations are suggested to consider PsyCap as the content for management programs and interventions (e.g., web-based training intervention devised by Luthans et al. (2008), new resource-based intervention programme (FAMILY intervention) developed by Costantini et al. (2017), thereby enhancing their PsyCap. However, we further found that the effectiveness of PsyCap depends on the level of climate for innovation. Thus, in addition to enhance PsyCap, organizations also should create high climate for innovation by taking some effective measures, such as practical and emotional support for creative efforts and organizational inducements for creative performance, job redesign and promoting employees’ job crafting behaviors (Wang and Rode, 2010; Petrou et al., 2018).

### Limitations and Future Directions

Several limitations should be noted in the study. First, the data were collected in a single organization in China, which may limit the generalizability of our findings to other industries or cultures. Future research should test whether the conclusions apply in other contexts. Second, our study relied on self-reported data, raising concerns about common method variance (CMV). Future research should focus on supervisory ratings of behavioral support for change to reduce CMV. Third, measures were collected at two time points, which may limit the explanation of the moderated mediation effect. Future research should conduct three waves of surveys to strengthen causal inference. Forth, our findings indicate that openness to change has a partial mediating role in the relationship between PsyCap and behavioral support for change. We thus encourage future research to investigate other relevant potential mediators, which may help explain the positive effect of PsyCap on behavioral support for change. Fifth, Table 2 showed that climate for innovation had a positive correlation with openness to change (*r* = *0.44, p* < *0.01*) and behavioral support for change (*r* = *0.39, p* < *0.01*), which suggest that climate for innovation may have direct effects on openness to change and behavioral support for change. Therefore, it is suggested that future research develop a theoretical model to explore their relationships.

## Data Availability Statement

The raw data supporting the conclusions of this article will be made available by the authors, without undue reservation.

## Ethics Statement

Ethical review and approval were not required for the study on human participants in accordance with the local legislation and institutional requirements. Written informed consent to participate in this study was provided by the participants.

## Author Contributions

The author confirms being the sole contributor of this work and has approved it for publication.

## Conflict of Interest

The author declares that the research was conducted in the absence of any commercial or financial relationships that could be construed as a potential conflict of interest.

## Publisher’s Note

All claims expressed in this article are solely those of the authors and do not necessarily represent those of their affiliated organizations, or those of the publisher, the editors and the reviewers. Any product that may be evaluated in this article, or claim that may be made by its manufacturer, is not guaranteed or endorsed by the publisher.
